# Simultaneous Multi-Ion Heavy Metal Sensing Using Pulse and Stripping Voltammetry at Functionalized Nanomaterial-Modified Glassy Carbon Electrodes

**DOI:** 10.3390/ijms27062586

**Published:** 2026-03-11

**Authors:** Aidyn Abilkas, Nargiz Kazhkenova, Bakhytzhan Baptayev, Robert J. O’Reilly, Mannix P. Balanay

**Affiliations:** 1 Department of Biology, Nazarbayev University, Astana 010000, Kazakhstan; aidyn.abilkas@nu.edu.kz; 2 Department of Chemistry, Nazarbayev University, Astana 010000, Kazakhstan; nargiz.kazhkenova@nu.edu.kz; 3 National Laboratory of Astana, Nazarbayev University, Astana 010000, Kazakhstan; bbaptayev@nu.edu.kz; 4 School of Science and Technology, University of New England, Armidale, NSW 2351, Australia

**Keywords:** glassy carbon electrode, heavy metal detection, nanomaterial-modified sensors, simultaneous multi-ion sensing, electrochemical sensing, environmental toxicology

## Abstract

Glassy carbon electrodes (GCEs) have gained increased attention for the sensitive electrochemical detection of heavy metals due to their excellent chemical stability, wide potential window, and good electrical conductivity. These characteristics make GCEs an effective platform for sensor development. In particular, nanomaterial-modified GCEs have emerged as a promising strategy, offering enhanced sensitivity, selectivity, and faster response compared to conventional analytical techniques. This review summarizes recent advances over the past five years in the use of GCEs modified with chemically synthesized nanoparticles for the simultaneous detection of multiple heavy metal ions, including cadmium, lead, mercury, and chromium. It also includes how quantum chemical methods have aided our understanding of these phenomena. Heavy metals pose significant environmental and public health risks, with well-documented neurological, cardiovascular, reproductive, and carcinogenic effects, highlighting the need for accurate and rapid monitoring methods. Regulatory limits established by organizations such as the World Health Organization and the Environmental Protection Agency further emphasize the demand for highly sensitive detection technologies. This review examines the fundamental properties of GCEs, common nanomaterial modification techniques, and their application in multi-ion detection systems. Key advantages such as cost-effectiveness, portability, and adaptability to diverse sample matrices are highlighted. Current challenges, including electrode fouling, selectivity, and matrix interference, are also addressed, along with future perspectives for improving GCE-based sensors for real-world environmental monitoring.

## 1. Introduction

The electrochemical detection of heavy metal ions using nanomaterial-modified electrodes has undergone substantial evolution over the past decade. Earlier reviews, including the comprehensive survey by Solangi and co-workers [1], summarized nanoparticle-based sensing platforms for environmental monitoring, primarily covering developments prior to 2020. While these studies provided valuable overviews of nanomaterial-assisted sensing strategies, most treated electrode materials broadly or focused predominantly on single-ion detection. Consequently, a critical synthesis that specifically examines recent material architectures engineered for simultaneous multi-ion electrochemical detection on glassy carbon electrodes (GCEs) remains limited.

**Figure 1:**
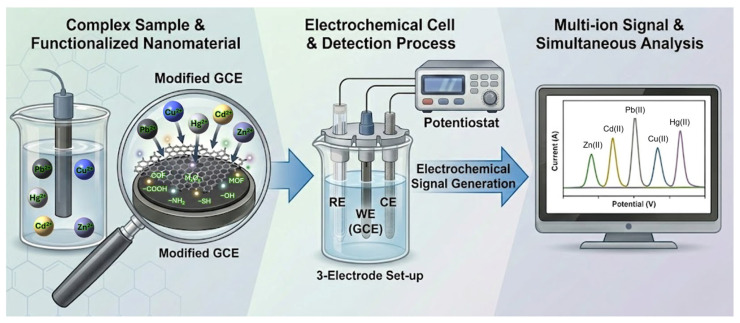
Schematic representation of the sensing pipeline, from complex sample acquisition to multi-analyte signal processing. The inset highlights the role of rational interface engineering, where specific functional groups are utilized to enhance the adsorption and electron-transfer characteristics of the modified glassy carbon electrode (GCE). Note that RE, WE, and CE represent reference, working, and counter electrodes, respectively.

Glassy carbon electrodes serve as chemically inert, mechanically robust, and electrochemically stable conductive substrates with wide potential windows and low background currents. However, the intrinsic electrochemical activity of bare GCEs toward heavy metal stripping is relatively modest. Analytical performance is dictated primarily by the physicochemical properties of the surface modifier rather than by the substrate itself [2,3,4]. The overall detection strategy for simultaneous multi-ion sensing involves modification of the GCE surface with functional nanomaterials to improve selective adsorption and electron transfer, enabling complex sample analysis and multi-ion voltammetric signal generation, as illustrated schematically in Figure 1. Therefore, recent progress in trace-level detection has been increasingly driven by rational interface engineering, in which nanoscale structure, surface chemistry, defect density, and functional group distribution are deliberately tailored to control adsorption, nucleation, and electron transfer processes.

Since 2020, research has increasingly shifted from single-component nanomaterials to multifunctional hybrid nanocomposites designed to better address the complexity of multi-metal systems [5,6,7,8,9]. These advanced platforms typically integrate metal or metal oxide nanoparticles such as bismuth, gold, zinc oxide, and magnetite (Fe_3_O_4_), along with graphene derivatives including reduced graphene oxide (rGO), carbon nanotubes (CNTs), metal–organic frameworks (MOFs), conductive polymers such as polyaniline and polypyrrole, and heteroatom-doped porous carbons [6,7,8,9,10,11].

The rationale behind such integration is synergistic coupling. Conductive carbon frameworks enhance charge-transfer kinetics, while metal nanoparticles facilitate alloy formation or catalytic stripping. MOFs and porous carbons provide high surface area and tunable coordination environments, enabling selective preconcentration of specific metal ions. Conductive polymers contribute functional groups that modulate local pH or binding affinity. Rather than serving merely as signal amplifiers, these materials actively regulate interfacial thermodynamics and kinetics, influencing metal ion accumulation efficiency, stripping peak separation, and resistance to fouling [7,10,12].

Simultaneous electrochemical detection introduces additional physicochemical complexities absent in single-ion systems. During anodic stripping voltammetry (ASV), co-deposited metals may form intermetallic compounds or compete for active nucleation sites, leading to distorted or overlapping peaks [8,13]. Competitive adsorption can suppress signals of trace-level ions when higher-concentration species dominate surface binding sites. Moreover, differences in diffusion coefficients and deposition potentials further complicate signal interpretation. Addressing these challenges requires deliberate structural design, including spatial confinement effects, heterojunction interfaces that modulate electron density distribution, defect engineering to create preferential nucleation centers, and hierarchical porosity to improve mass transport [9,10,11,12].

Among electroanalytical techniques, ASV remains the dominant method for multi-metal quantification due to its intrinsic preconcentration capability and compatibility with trace analysis [13]. However, recent progress demonstrates that performance improvements arise not solely from enhanced conductivity, but from controlled adsorption–desorption equilibria, localized coordination chemistry, and regulation of metal nucleation/stripping behavior at the interface [7,10,12].

Recent progress in multi-ion electrochemical heavy-metal sensing is increasingly driven by electrode design choices that deliberately control interfacial chemistry and transport rather than by “nanomaterial novelty” alone. In particular, studies on non-conductive modifiers emphasize that performance improvements can arise from adsorption-mediated preconcentration, nucleation/stripping behavior, and local micro-environment effects at the interface, even when the modifier itself is not highly conductive, highlighting the need for structure–performance relationships supported by advanced characterization and theory [13].

Despite rapid growth in publications, several gaps remain. First, many studies report ultra-low detection limits but provide limited mechanistic insight into peak separation mechanisms in multi-ion systems. Second, comparative evaluation across different composite classes is scarce, making it difficult to identify universal design principles. Third, systematic discussions of how hybrid architectures mitigate intermetallic interference remain fragmented. As a result, the field lacks a cohesive framework linking nanomaterial structure to multi-ion electroanalytical performance.

This review addresses current limitations in the electrochemical detection of heavy metal ions by critically analyzing chemically synthesized nanomaterials integrated onto glassy carbon electrode platforms for simultaneous multi-ion detection, with a focus on literature published between 2020 and 2026. Rather than merely compiling detection limits, the discussion emphasizes key aspects of material design strategies for GCE modification, mechanistic insights into signal discrimination and interference suppression, and the synergistic effects observed in hybrid nanocomposites. Additionally, emerging structure–performance correlations are highlighted, supported by advanced characterization techniques and theoretical modeling, providing a comprehensive perspective on the rational design of nanomaterial-based GCE sensors.

By concentrating specifically on interfacial engineering approaches that enable high-resolution, trace-level multi-metal quantification, this review aims to clarify design principles for next-generation electrochemical sensors. Such insight is essential for developing robust, reproducible, and application-oriented platforms capable of operating reliably in complex environmental matrices.

## 2. Theoretical and Mechanistic Foundations of Electrochemical Multi-Ion Heavy Metal Detection

### 2.1. Interfacial Redox Principles in Heavy Metal Electroanalysis

Electrochemical detection of heavy metal ions in aqueous environments is fundamentally governed by interfacial redox reactions occurring at the electrode–solution boundary. These techniques offer high sensitivity, low detection limits, rapid response times, and compatibility with portable instrumentation [12,14,15,16]. In contrast to bulk spectroscopic approaches, electrochemical methods directly interrogate charge-transfer events at the interface, where the analytical signal is generated. As outlined in the general analytical workflow (Figure 1), the measured current arises from a sequence of interfacial processes rather than a single redox event. A mechanistic understanding of these steps is therefore essential to explain both sensitivity and selectivity.

**Figure 2:**
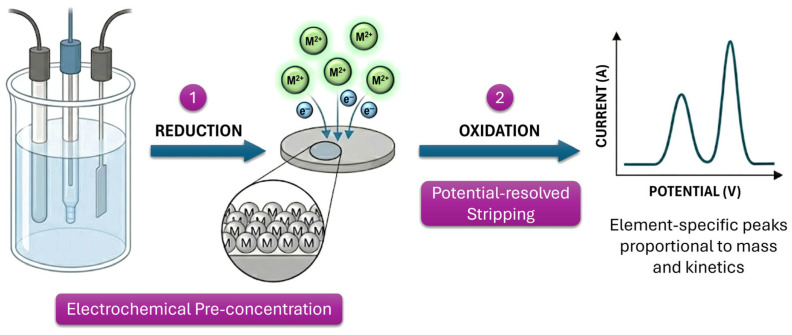
Detailed visualization of the two-step electrochemical detection mechanism: (1) Cathodic pre-concentration, involving the reduction and accumulation of metal ions onto the electrode surface, and (2) Anodic stripping, where potential-resolved oxidation generates element-specific current peaks. The peak morphology serves as a diagnostic tool for both analyte concentration and interfacial charge-transfer kinetics.

Figure 2 conceptually separates this interfacial sequence into two principal kinetic stages: (i) electrochemical pre-concentration and (ii) potential-resolved stripping. During the pre-concentration step, dissolved metal ions are electrochemically reduced and accumulated onto the electrode surface. This accumulation step effectively amplifies the analyte signal by increasing the local surface concentration. In the subsequent stripping step, the deposited metals are re-oxidized, generating element-specific current peaks. The peak current is proportional to the accumulated mass, while the peak potential reflects the thermodynamic redox potential and interfacial coordination environment. The magnitude and position of stripping peaks are therefore governed by a combination of thermodynamic and kinetic parameters, including formal redox potentials, adsorption energetics, nucleation behavior, and interfacial electron-transfer rates. In multi-ion systems, the analytical resolution depends strongly on differences in these parameters. Rational interface engineering, through material selection, surface functionalization, or nanostructuring, enables deliberate modulation of these thermodynamic and kinetic factors, thereby improving peak separation and detection performance.

A defining strength of voltammetric techniques in multi-metal analysis lies in their discrimination within the potential domain: each metal ion ideally produces a characteristic stripping peak corresponding to its redox thermodynamics and coordination chemistry. However, in realistic environmental matrices, interfacial phenomena such as competitive adsorption, surface site saturation, nucleation competition, and intermetallic compound formation frequently distort peak positions and broaden signals. These coupled processes can reduce peak resolution and compromise analytical accuracy. Consequently, surface chemistry and interfacial structure become decisive determinants of sensitivity, selectivity, and reproducibility in heavy metal electroanalysis [12,14,15,16].

### 2.2. Pulse and Stripping Voltammetry for Multi-Ion Detection

Pulse voltammetric techniques such as square-wave voltammetry (SWV) and differential pulse voltammetry (DPV) enhance analytical sensitivity by suppressing capacitive background currents and amplifying faradaic responses [17,18,19]. In stripping voltammetric techniques, including square-wave anodic stripping voltammetry (SWASV) and differential pulse anodic stripping voltammetry (DPASV), pulsed excitation is combined with controlled deposition to achieve ultra-trace detection limits [20,21,22,23].

Instrumental parameters, including pulse amplitude, frequency, step potential, deposition potential, and accumulation time, directly influence peak sharpness, symmetry, and the signal-to-noise ratio. Although waveform optimization enhances analytical clarity and improves measurement precision, it does not fundamentally modify adsorption thermodynamics or interfacial selectivity. Consequently, in multi-ion systems, instrumental refinement should be integrated with rational interface design to ensure reliable peak discrimination and accurate analytical performance. Table 1 provides a summary of the operational principles, analytical characteristics, and comparative advantages of these techniques.

### 2.3. Role of Supporting Electrolytes and Buffer Systems

The supporting electrolyte critically governs electrochemical behavior across both stripping and non-stripping voltammetric techniques. In anodic stripping voltammetry, acetate buffer systems (typically pH 4.0–5.5) are widely employed because they maintain metal ions in soluble form, suppress hydrolysis and precipitation, stabilize peak profiles, and promote reproducible deposition–stripping processes [24]. Slightly acidic conditions facilitate efficient metal reduction while preserving signal stability.

Beyond stripping methods, base electrolytes such as KCl, KNO_3_, Na_2_SO_4_, and phosphate buffers significantly influence cyclic voltammetry (CV), DPV, and related techniques. Supporting electrolytes regulate ionic strength, solution conductivity, and migration suppression while defining the structure of the electrical double layer at the electrode interface. Variations in electrolyte identity and pH alter metal ion speciation, complex formation equilibria, adsorption behavior, and stripping potentials. For example, chloride-containing media may induce metal–chloride complex formation that shifts peak potentials and affects resolution, whereas nitrate-based systems provide electrochemical inertness but limited buffering capacity [24,25].

In multi-ion environments, electrolyte composition becomes a mechanistic control parameter influencing competitive adsorption, deposition kinetics, and peak overlap. Despite its central role, systematic cross-comparative evaluation of electrolyte-dependent multi-metal discrimination remains relatively underreported.

### 2.4. Nanomaterial-Modified Glassy Carbon Electrodes: Interface Engineering Strategies

Given the dominant influence of interfacial phenomena, nanomaterial-modified glassy carbon electrodes have become central to heavy metal sensing research. GCEs provide chemical stability, a wide potential window, and compatibility with diverse functionalization strategies. Incorporation of metal–organic frameworks (MOFs), covalent organic frameworks (COFs), graphene and carbon nanotubes, metal and metal oxide nanoparticles, and conductive polymers enhances active surface area, introduces chemically addressable coordination sites, and improves electrical conductivity [16,22,26,27,28,29,30,31,32,33,34,35,36,37,38,39,40,41,42,43,44,45,46,47,48,49,50,51,52,53,54,55,56,57,58,59,60,61,62,63,64,65,66,67,68,69,70,71,72,73,74,75,76,77,78,79,80,81,82,83,84,85,86,87,88,89].

For modified GCEs, analytical performance is governed by the interplay between preconcentration thermodynamics, charge-transfer kinetics, and multi-ion discrimination efficiency. Preconcentration efficiency depends on adsorption energy, coordination geometry, porosity, and active site density. Charge-transfer kinetics are influenced by conductivity, defect density, and electronic band structure. Peak discrimination in multi-ion systems is controlled by differences in nucleation behavior, competitive binding equilibria, and deposition–stripping energetics.

Although nanomaterial modification significantly improves sensitivity, challenges such as electrode fouling, surface passivation, and matrix interference persist. Thus, performance enhancement requires not only increased surface area but also controlled interfacial electronic structure.

### 2.5. Quantum Mechanical and Data-Driven Insights into Metal–Interface Interactions

At the atomic scale, electrochemical sensitivity in heavy metal detection is primarily governed by the interactions between metal ions and surface functional groups on the electrode. Density functional theory (DFT) and related quantum mechanical methods provide mechanistic insight into these interactions by quantifying adsorption energies, coordination geometries, and interfacial charge redistribution. For instance, Flórez and colleagues [89] demonstrated that oxygen-containing groups on carbon surfaces enable spontaneous adsorption of Cd(II), Pb(II), and Ni(II), highlighting that surface oxygen functionalities enhance orbital overlap between metal ions and carbon frameworks, thereby stabilizing preconcentrated ions. Similarly, Hernández-Fernández et al. [90] showed that thiol-functionalized graphene quantum dots preferentially bind Pb(II) and Cd(II) due to optimized frontier orbital alignment, indicating that heteroatom chemistry directly modulates adsorption strength and selectivity.

Graphene–carbene composites have also been shown to exhibit strong interaction energies with Cd(II), Hg(II), and Pb(II), as reported by Baachaoui and associates [91], suggesting that introducing covalently bound active sites can enhance ion preconcentration while maintaining electronic conductivity. Song and collaborators [92] emphasized that the electronic structure of the modifier, rather than surface area alone, determines stripping peak magnitude, underlining that orbital hybridization and local density-of-states at the interface dictate interfacial electron-transfer kinetics. Notably, Shen and co-authors [93] provided experimental validation of DFT predictions in boron-modified bio-carbon electrodes, showing enhanced simultaneous detection of Cd(II), Pb(II), and Cu(II), which confirms that computational descriptors such as adsorption energy and charge transfer can reliably predict real-world electrode performance.

**Figure 3:**
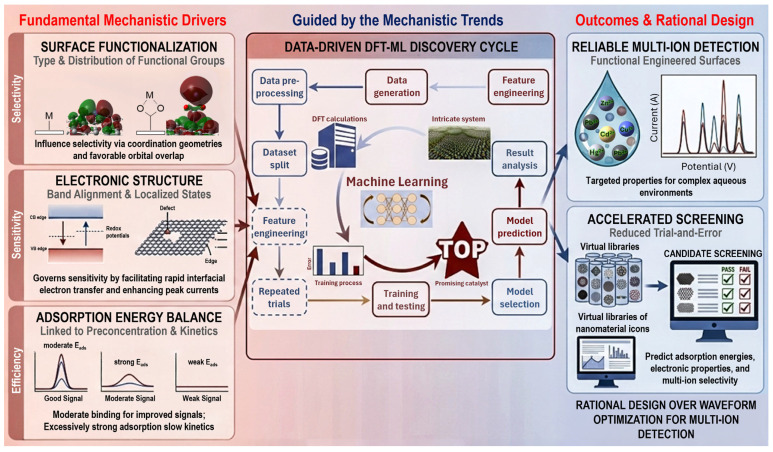
Schematic representation of quantum mechanical and data-driven design principles for electrochemical heavy metal detection. Surface functionalization and electronic structure govern ion selectivity and interfacial charge transfer, while adsorption energy balance determines preconcentration efficiency. Density functional theory (DFT) calculations generate mechanistic descriptors that feed into machine learning (ML) models for accelerated screening and rational design of multi-ion selective electrode materials. The data-driven DFT-ML discovery cycle was taken from reference [94]. Copyright (2025) the authors, some rights reserved; exclusive licensee Elsevier. Distributed under a Creative Commons Attribution 4.0 International License (CC BY 4.0).

Collectively, these studies reveal several important mechanistic trends. The type and distribution of surface functional groups strongly influence selectivity by stabilizing particular metal ions through favorable orbital overlap and coordination geometries. Electronic structure, including band alignment and localized states at edges or defects, governs sensitivity by facilitating rapid interfacial electron transfer and enhancing peak currents. The efficiency of preconcentration is closely linked to adsorption energy: moderate to strong binding improves stripping signals, but excessively strong adsorption can slow kinetics and broaden peaks, potentially reducing analytical resolution. Beyond traditional DFT, machine learning models trained on DFT-derived datasets [94] have emerged as powerful tools for rapidly predicting adsorption energies, electronic properties, and multi-ion selectivity, enabling accelerated screening of candidate nanomaterials with reduced experimental trial-and-error. The mechanistic relationships among surface functionalization, electronic structure, adsorption energetics, and machine-learning-guided material screening are schematically illustrated in Figure 3. The figure integrates fundamental mechanistic drivers, surface chemistry, band structure, and adsorption energy balance, with a data-driven DFT–ML discovery cycle, highlighting how computational descriptors inform accelerated virtual screening and rational design of multi-ion selective electrode materials.

Overall, quantum mechanical and data-driven approaches provide predictive insight into the fundamental factors controlling electrochemical sensitivity, selectivity, and interfacial kinetics. The collective findings indicate that rational electrode design should prioritize functionally engineered surfaces with tailored electronic properties, rather than relying solely on increased surface area or waveform optimization, to achieve reliable multi-ion heavy metal detection in complex aqueous environments.

### 2.6. Mechanisms of Cross-Talk and Strategies for Multi-Ion Resolution

In multi-ion electrochemical systems, analytical limitations often arise not from insufficient sensitivity but from inter-ion interference phenomena collectively referred to as cross-talk. Unlike single-analyte detection, where adsorption and stripping occur independently, multi-metal environments involve simultaneous competition for active sites, overlapping reduction potentials, and coupled nucleation processes. These interactions introduce both thermodynamic and kinetic complexities that distort stripping responses and reduce quantitative reliability, particularly in anodic stripping voltammetry and pulse-based stripping techniques [17,18,19,20,21,22,23].

Systematic investigations of interference mechanisms in multi-metal ASV systems have shown that competitive adsorption is a primary factor governing peak suppression and signal attenuation [95]. When two or more ions possess comparable reduction potentials, preferential adsorption of one species can inhibit the deposition efficiency of others, even at similar bulk concentrations. Thermodynamic modeling studies further demonstrate that the relative adsorption free energies of metal ions on nanostructured surfaces determine the severity of these interference effects [96]. In addition to adsorption competition, co-deposition and intermetallic alloy formation represent critical sources of peak distortion. Experimental analyses of simultaneous metal detection have confirmed that alloy formation between species such as Cd, Pb, and Cu can shift stripping potentials and broaden peak profiles, thereby complicating quantitative interpretation [95].

Targeted interface engineering has been shown to mitigate such effects. For example, studies employing amino acid–functionalized glassy carbon electrodes demonstrated improved discrimination among Zn(II), Cd(II), Cu(II), and Hg(II) due to selective coordination interactions that reduce competitive adsorption [97]. Similarly, nanostructured materials with hierarchical porosity have been reported to enhance ion accumulation while spatially distributing active sites, thereby minimizing direct site competition and improving charge-transfer efficiency in multi-ion environments [98]. Complementary signal-processing strategies, including baseline correction and peak deconvolution algorithms, have also been applied to resolve partially overlapping stripping peaks in complex matrices without modifying the underlying electrode chemistry [99].

Kinetic factors further contribute to cross-talk behavior. Differences in electron-transfer rates and nucleation kinetics can cause one ion to dominate the faradaic response even at lower concentration, while surface restructuring during deposition may dynamically alter active site availability. Integrated theoretical–experimental studies confirm that selective binding motifs reshape adsorption energetics upstream, thereby reducing downstream peak overlap during stripping [13].

Overall, cross-talk in multi-ion electrochemical detection arises from coupled thermodynamic and kinetic interactions at the electrode–solution interface. Competitive adsorption, co-deposition, and alloy formation distort stripping signals and complicate quantification. Effective mitigation therefore requires coordinated control of surface chemistry, nanostructure architecture, deposition protocols, and signal interpretation to achieve reliable simultaneous heavy metal monitoring.

## 3. Applications of Glassy Carbon Electrodes in Heavy Metal Detection

Glassy carbon electrodes are widely employed in electrochemical heavy metal sensing owing to their broad potential window, low background current, excellent electrical conductivity, and chemical stability. While unmodified GCEs provide a reliable electrochemical substrate, their analytical performance is significantly enhanced through rational surface modification. The rapid development of multifunctional and hybrid modifiers has enabled simultaneous detection of multiple heavy metal ions with improved sensitivity and selectivity. As these architectures become increasingly complex, traditional classifications based purely on material type (e.g., graphene-based, metal oxide-based) are no longer sufficient to describe structure–function relationships within sensor systems.

To provide clearer insight into performance enhancement strategies, recent advances in GCE-based heavy metal sensors are organized here according to the primary functional role of the surface modifier in the sensing process rather than by composition alone. This functional classification emphasizes how modifiers improve electrochemical behavior by acting as conductive scaffolds, ion-coordination platforms, electrocatalytic centers, or bio-inspired recognition interfaces. Such a perspective highlights synergistic interactions within hybrid materials, where multiple mechanisms operate concurrently to enhance signal amplification and multi-ion discrimination. Particular attention is given to platforms capable of simultaneously detecting Hg(II), Pb(II), Cd(II), Cu(II), and Cr(VI) in aqueous systems. For consistency across studies, detection limits are reported in nanomolar (nM) units. The reviewed systems are therefore discussed within four major categories: (1) carbon-based conductive scaffolds, (2) porous coordination frameworks, (3) metallic and metal oxide electrocatalysts, and (4) bio-inspired and polymeric interfaces.

### 3.1. Carbon-Based Conductive Scaffolds

Carbon-based conductive scaffolds form the structural and electrical foundation of most high-performance electrochemical sensors for heavy metal detection. Comparative studies across carbon nanotubes (CNTs), graphene derivatives, graphitic carbon nitride (g-C_3_N_4_), graphdiyne, and MXenes show that, despite differences in dimensionality and surface chemistry, their primary contribution lies in establishing continuous electron-transport pathways that enable efficient signal transduction at the electrode–electrolyte interface [22,26,44,45,46,47,48,49,50,51,52,53,54,55,56]. As a result, sensing performance is governed less by the intrinsic chemical activity of the scaffold itself and more by how effectively the carbon framework maintains electrical continuity while supporting secondary functional components (Table 2).

**Figure 4:**
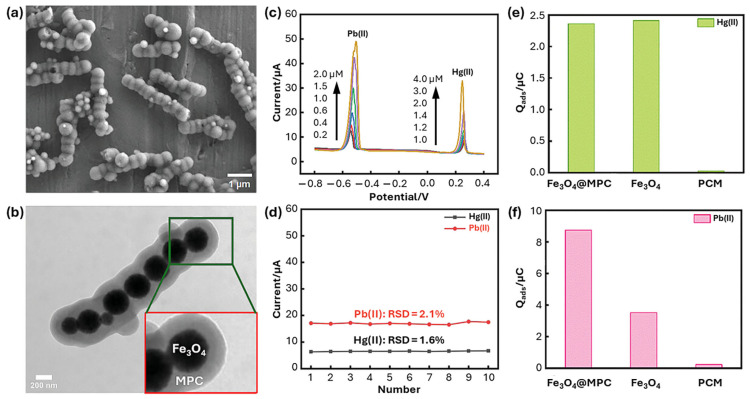
(**a**) SEM and (**b**) TEM images of Fe_3_O_4_@MPC-2, illustrating the core-shell structure. (**c**) DPASV curves and (**d**) repeatability graph of Fe_3_O_4_@MPC-2-modified GCE for the simultaneous detection of multiple metal ions. (**e**) Comparison of the adsorbed amounts (Q_ads_) of Hg(II) and (**f**) Pb(II) on different electrodes. Note: MPC and PCM denote mesoporous carbon nanochains and pure carbon materials, respectively. Adapted with permission from reference [49]. Copyright (2023) the authors, some rights reserved; exclusive licensee Wiley-VCH. Distributed under a Creative Commons Attribution 4.0 International License (CC BY 4.0).

One-dimensional CNT networks and two-dimensional graphene-based scaffolds represent the two most widely adopted conductive architectures, each offering distinct advantages. CNT-based scaffolds, particularly multi-walled CNTs, form interconnected three-dimensional networks that facilitate rapid electron percolation and efficient mass transport, leading to high stripping currents and robust signal stability [22]. In contrast, graphene and reduced graphene oxide (rGO) provide extended planar conduction pathways with low intrinsic resistance, which are particularly effective at suppressing background currents and enabling simultaneous multi-metal detection [45,46,47]. These comparisons indicate that CNT scaffolds favor current amplification through network connectivity, whereas graphene-based scaffolds emphasize charge-transfer uniformity and low noise. An example of how graphene-based scaffolds increases the sensitivity and stability of the electrode is presented in Figure 4.

The importance of conductive scaffolds becomes especially evident when comparing intrinsically conductive carbons with semiconducting or poorly conductive materials. Carbon frameworks such as graphene or CNTs consistently enhance the electrochemical performance of g-C_3_N_4_, layered double hydroxides, and metal oxides by compensating for their limited electrical conductivity [26,44,45,49,50]. In these hybrids, the carbon scaffold dominates electron transport, while the secondary phase contributes chemical affinity or catalytic activity. Without the carbon backbone, these materials exhibit sluggish kinetics and diminished sensitivity, underscoring the central role of carbon scaffolds in enabling effective signal transduction.

Surface functionalization further differentiates carbon scaffolds without compromising their conductive role. Functionalized CNTs (e.g., MWCNT-COOH) preserve network conductivity while introducing coordination sites that promote analyte accumulation, whereas defect-rich rGO offers abundant anchoring sites for polymers, metal nanoparticles, and oxides with minimal disruption to electron flow [22,46,47]. Comparative results suggest that scaffolds with high defect tolerance, such as rGO, provide greater architectural flexibility, while CNT networks maintain superior mechanical and electrical stability under repeated electrochemical cycling.

Ionic liquid–carbon systems highlight the structural dominance of carbon scaffolds in maintaining charge transport. In BMIMPF_6_–MWCNT electrodes, the ionic liquid improves ion mobility and interfacial wetting, but the CNT framework ensures electrical continuity and signal reproducibility [22]. This comparison reinforces the concept that secondary components modulate interfacial processes, whereas carbon scaffolds govern overall electron-transfer efficiency.

Beyond conventional CNT and graphene scaffolds, emerging carbon allotropes expand the design space for conductive architectures. MXene–carbon hybrids, such as Ti_3_C_2_T_x_–rGO, rely on rGO to prevent MXene restacking and preserve continuous electron pathways, enabling high sensitivity at low analyte concentrations [52]. Structurally engineered carbons, including electrochemically reduced graphene oxide, heteroatom-doped carbon nitride/graphene hybrids, and graphdiyne, demonstrate that tailored carbon frameworks alone can deliver high conductivity, structural stability, and rapid electrochemical response without extensive secondary modification [54,55,56].

Overall, comparative evaluation of carbon-based conductive scaffolds reveals that sensor performance is dictated by scaffold dimensionality, network connectivity, and defect tolerance rather than the specific carbon allotrope employed. By providing electrically continuous backbones that suppress background interference and support efficient interfacial charge transfer, carbon-based scaffolds remain the cornerstone of advanced electrochemical platforms for heavy metal detection.

### 3.2. Porous Frameworks for Ion Coordination

Metal–organic frameworks, zeolitic imidazolate frameworks (ZIFs), and covalent organic frameworks have emerged as highly effective modifiers for glassy carbon electrodes, primarily due to their tunable porosity, high surface area, and abundant coordination sites. A defining advantage of these porous frameworks is their ability to function as molecular sieves, selectively capturing and preconcentrating target metal ions within well-defined pore environments [27]. This preconcentration effect significantly amplifies electrochemical signals and improves detection sensitivity, particularly at trace and ultra-trace concentration levels. Representative examples of these frameworks and their target analytes are summarized in Table 3.

MOFs are especially attractive because their metal nodes and organic linkers can be rationally selected to tailor pore size, surface chemistry, and redox activity. Bimetallic MOFs, in particular, offer synergistic catalytic behavior that enhances electrochemical responsiveness. For example, Fe–Co MOF hybrids integrated with functionalized multiwalled carbon nanotubes (Fe–Co–MOF@MWCNT–COOH/GCE) [27] exploit both the redox-active metal centers and the conductive carbon scaffold, enabling efficient ion adsorption and accelerated electron transfer. This architecture yields low-nanomolar detection limits for Cd(II) and Pb(II), illustrating how MOFs simultaneously act as ion reservoirs and electrocatalytic platforms. Similarly, carboxyl-functionalized MIL-101(Cr) combined with MWCNTs benefits from hierarchical porosity and strong metal–ligand interactions, facilitating the simultaneous detection of Pb(II), Cu(II), and Hg(II) with high sensitivity [29].

**Figure 5:**
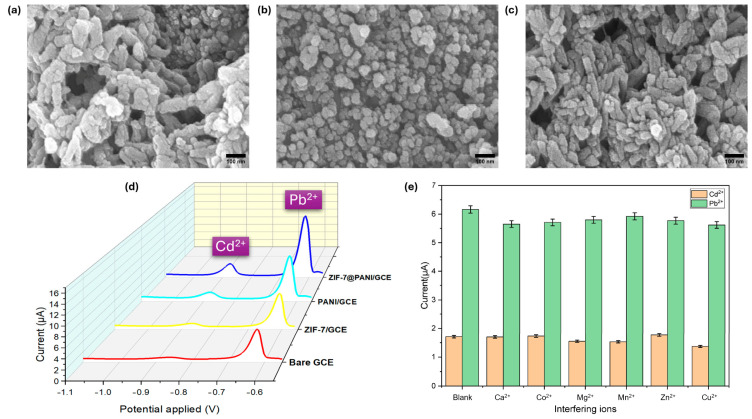
SEM images of (**a**) PANI, (**b**) ZIF-7, and (**c**) ZIF-7@PANI modified electrodes. (**d**) DPV curve of the different electrodes in 0.1 M acetate buffer (pH 5) with 10 µM mixture of Cd(II) and Pb(II). (**e**) Selectivity of the ZIF-7@PANI/GCE sensor for 5 μM Cd(II) and Pb(II) in the presence of 50 μM interfering ions. Adapted with permission from reference [28]. Copyright (2025) the authors, some rights reserved; exclusive licensee MDPI. Distributed under a Creative Commons Attribution 4.0 International License (CC BY 4.0).

ZIFs represent a distinct subclass of MOFs that combine zeolite-like topology with metal–imidazolate coordination, offering exceptional chemical stability and size-selective ion transport. Their well-defined microporous channels enable selective ion sieving, while surface functionalization further enhances affinity toward specific analytes. ZIF-7@PANI exemplifies this concept by integrating a conductive polymer with the ZIF, improving charge transport while maintaining efficient ion preconcentration, resulting in sub −10 nM detection limits for Cd(II) and Pb(II) [28] as shown in Figure 5. ZIF-67-based composites, including ZIF-67/GO [32] and La-doped ZIF-67@L-Cys [33], leverage the adsorption capacity of the cobalt-based framework in conjunction with functional groups from graphene oxide or amino acids, enabling simultaneous detection of Pb(II), Hg(II), Zn(II), and Cr(III). In parallel, ZIF-8 hybrids such as Hg/CMWCNTs@ZIF-8 [30] and Bi@ZIF-8/CMWCNTs [31] demonstrate how the incorporation of electroactive metals into ZIF pores further refines ion selectivity and signal stability, reinforcing the molecular sieve behavior of these frameworks.

COFs offer a complementary approach to ion coordination by relying entirely on covalent bonding, resulting in lightweight, crystalline, and chemically robust porous networks. Their ordered π-conjugated structures facilitate charge transport, while heteroatom-rich linkages provide strong binding sites for metal ions. COFs such as TPT-COF [41], and SNW1 [42] exhibit exceptional stability in aqueous media and maintain high adsorption capacities for Pb(II) and Cd(II), even in complex matrices. Two-dimensional COFs like COFBTLP-1 [40] further enhance accessibility to active sites, enabling efficient ion diffusion and ultra-low detection limits down to the sub-nanomolar range for Cd(II), Pb(II), Cu(II), and Hg(II). These characteristics underscore the ability of COFs to combine molecular sieving, structural stability, and electrochemical activity within a single platform.

Beyond these core classes, hybrid porous frameworks continue to expand design flexibility. Dendrimer-modified systems such as PAMAM/Ni-MOF [34] introduce dense chelating functionalities that strengthen metal–ligand interactions, significantly improving binding affinity toward Pb(II) and Cu(II). Likewise, Cr-BDC MOFs [39] demonstrate how framework composition alone can deliver sub-nanomolar sensitivity for Cd(II), Pb(II), and Hg(II) through efficient ion confinement and preconcentration.

Overall, MOFs, ZIFs, and COFs serve not merely as passive supports but as active molecular sieves that govern ion selectivity, accumulation, and transport at the electrode interface. When coupled with conductive matrices or functional modifiers, these porous frameworks provide a highly tunable and powerful strategy for designing next-generation electrochemical sensors capable of sensitive, selective, and multiplexed heavy metal ion detection.

### 3.3. Metallic and Metal Oxide Electrocatalysts

Metal and metal oxide-integrated systems represent a central class of glassy carbon electrode modifiers for heavy metal detection due to their exceptional electrocatalytic properties. Their performance stems from a combination of intrinsic characteristics, including high redox activity, chemical stability, and strong affinity for heavy metal ions. When incorporated into GCEs, these materials enhance electron transfer kinetics, expand the electroactive surface area, and promote selective analyte adsorption which are all critical factors for achieving sensitive and reliable electrochemical detection. The benefits of these systems are further amplified when combined with conductive supports, carbon-based nanostructures, or polymeric matrices, producing hybrid platforms that synergistically combine high sensitivity, selectivity, and structural stability [99]. Table 4 summarizes representative examples of metallic and metal oxide modifiers used for the simultaneous detection of toxic heavy metals.

**Figure 6:**
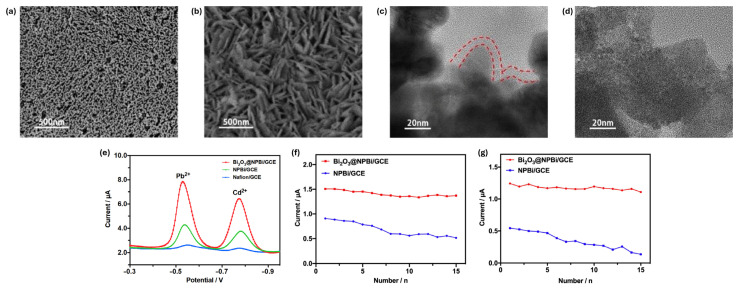
SEM and HRTEM images of (**a**,**c**) Bi_2_O_3_@NPBi and (**b**,**d**) NPBi. The HRTEM image of Bi_2_O_3_@NPBi shows a uniformly coated amorphous Bi_2_O_3_ layer on the NPBi surface. (**e**) SWASV responses of different electrodes in 200 µg L^−1^ Pb(II) and Cd(II) solutions using 0.1 M acetate buffer. Detection reproducibility of (**f**) Pb(II) and (**g**) Cd(II) across different electrodes. Adapted with permission from reference [64]. Copyright (2022) the authors, some rights reserved; exclusive licensee Elsevier. Distributed under a Creative Commons Attribution 4.0 International License (CC BY 4.0).

Bismuth-based systems exemplify the unique advantages of metal-integrated electrodes. Bismuth is particularly valuable because it can form “fused alloys” with heavy metals such as cadmium and lead, significantly enhancing stripping signals during electrochemical analysis. For example, the Bi/carboxyphenyl-modified GCE (Bi/CP/GCE) [57] integrates high-surface-area carbon paper to support the formation of bismuth alloys, which increases preconcentration efficiency and strengthens electrochemical signals. Similarly, the bismuth film GCE (BiFGCE) [60] relies on in situ formation of Bi–metal alloys during the preconcentration step, leading to markedly improved sensitivity. Advanced architectures, such as Bi_2_O_3_-decorated nanoporous bismuth (Bi_2_O_3_@NPBi) [64], fabricated through dealloying, feature a nanoporous bismuth framework coated with an amorphous Bi_2_O_3_ layer (Figure 6). This structure not only increases the electroactive surface area but also provides numerous active sites for selective adsorption of heavy metal ions, enhancing both sensitivity and resistance to interference. Mechanistically, these bismuth-based systems excel because alloy formation facilitates electron transfer, while the high surface area and chemical affinity of Bi for metal ions enable efficient preconcentration, resulting in ultralow detection limits often below 1 nM which is ideal for trace-level detection in environmental and biomedical contexts [59,60,61,62,63,64,65].

In addition to bismuth, noble metal nanoparticles such as gold and silver play a pivotal role in heavy metal sensing. These metals contribute strong electrocatalytic effects and can form alloys or amalgams with target heavy metals, amplifying stripping responses. For example, Ag nanoparticles enhance signal intensity through both catalytic activity and alloy formation with Cd(II), Pb(II), Cu(II), and Hg(II) [71,76], while Au nanoparticles facilitate rapid electron transfer and improve the selectivity of multi-metal detection.

Metal oxides such as Ag_2_CrO_4_, NiO, Co_3_O_4_, and CoFe_2_O_4_ further expand the toolkit for electrocatalytic enhancement. These oxides provide redox-active surfaces that accelerate electrochemical reactions and often exhibit strong adsorption of specific metal ions. Hybrid systems combining metal oxides with carbon nanostructures which include GO, rGO, or MWCNTs leverage the high conductivity and surface area of carbon materials alongside the catalytic properties of oxides. Representative examples include Co_3_O_4_/GO [68] and Co_3_O_4_/rGO [66], as well as chitosan-encapsulated cobalt ferrite nanoparticles (CoFe_2_O_4_@CTS) [69]. In CoFe_2_O_4_@CTS, the chitosan matrix provides abundant chelating sites for selective binding of heavy metal ions, while embedded CoFe_2_O_4_ nanoparticles catalyze electron transfer during redox reactions. This dual functionality, selective adsorption coupled with catalytic enhancement, dramatically improves sensitivity and selectivity.

The transition from binary to ternary nanocomposites represents a strategic shift in GCE modification. For instance, the synergy between rGO and metal oxides is not merely additive in terms of surface area. Rather, the carbon scaffold provides the long-range electronic conductivity necessary for rapid electron transfer, while the metal oxide dopants act as localized ‘ion-traps’ via specific coordination chemistry [99]. However, a recurring challenge in these architectures is the trade-off between catalyst loading and surface fouling. High-impact studies now suggest that the morphological control of the modifier, such as the use of hierarchical porous structures, is more critical for simultaneous detection than high mass-loading, as it ensures unhindered diffusion pathways for different ionic radii during the stripping step.

Beyond composition, structural engineering of these materials also plays a critical role. Porous or hierarchical architectures, such as coral-like L-cysteine/Ag@MnO_2_ (L-Cys/Ag@MnO_2_) hybrids [75], maximize the density of electrochemically active sites and facilitate rapid diffusion of analyte ions to the electrode surface. Such designs achieve ultralow detection limits (e.g., 0.052 nM for Pb(II) and 0.065 nM for Cd(II)), demonstrating that rational electrode architecture, combined with judicious choice of metal nanoparticles and oxides, is key to optimizing heavy metal sensor performance.

In summary, the integration of metallic nanoparticles (Bi, Au, and Ag) and metal oxides (Ag_2_CrO_4_, NiO, and Co_3_O_4_) into GCEs provides a multifaceted strategy for enhancing electrochemical detection [43,69,76]. The combination of alloy formation, electrocatalysis, high surface area, and selective adsorption establishes these materials as indispensable components in the design of ultrasensitive and selective heavy metal sensors.

### 3.4. Bio-Inspired and Polymeric Interfaces

Polymer- and biofunctionalized interfaces constitute one of the most versatile and effective classes of modifiers for electrochemical sensing platforms, owing to their tunable chemical functionality, structural flexibility, and intrinsic affinity toward heavy metal ions. Unlike purely inorganic modifiers, these materials enable targeted chemical or biological recognition, allowing simultaneous enhancement of sensitivity, selectivity, and signal stability. As a result, they are particularly well suited for trace- and ultratrace-level detection of toxic metal ions in complex matrices.

Conductive polymers such as polyaniline (PANI) and poly(1,2-diaminoanthraquinone) (PDAAQ) play a dual role in electrochemical sensors [77,84,85]. First, their redox-active backbones facilitate rapid electron transfer between the electrode surface and the target analyte, thereby improving signal transduction efficiency. Second, polymers like PANI significantly enhance the mechanical integrity and electrochemical stability of the sensing interface. The high ionic conductivity and environmental stability of PANI, combined with its protonation–deprotonation behavior, allow it to operate effectively across a wide pH range while maintaining reproducible electrochemical responses. These features make conductive polymers particularly attractive as interfacial layers in sensors designed for long-term or repetitive use.

In contrast, functional polymers and bio-inspired modifiers, including chitosan, L-cysteine (L-Cys), aptamers, and poly(amidoamine) (PAMAM) dendrimers, primarily contribute through their high density of specific binding sites [35,86]. Small-molecule modifiers such as L-cysteine provide strong and selective coordination with soft metal ions via thiol (–SH), amine (–NH_2_), and carboxyl (–COOH) groups. This targeted chemical recognition enables efficient preconcentration of metal ions such as Cd(II), Pb(II), and Hg(II) at the electrode–electrolyte interface, even in the presence of competing species. Similarly, PAMAM dendrimers offer a three-dimensional, highly branched architecture with a large number of terminal functional groups, enabling multivalent binding interactions and exceptional metal-ion capture efficiency.

The integration of polymeric or biofunctional modifiers with carbon nanostructures or metal-based nanoparticles has led to a new generation of hybrid sensing platforms with markedly enhanced analytical performance. Carbon nanomaterials contribute high surface area and excellent electrical conductivity, while metal or metal-oxide nanoparticles introduce catalytic activity and favorable redox kinetics. Within these hybrids, the polymeric matrix serves multiple functions: it stabilizes dispersed nanostructures, suppresses aggregation, improves interfacial adhesion, and introduces selective binding sites for metal ions. This synergistic combination results in lower detection limits, improved reproducibility, and enhanced long-term operational stability. Moreover, the use of naturally derived biopolymers supports the development of environmentally benign and sustainable electrochemical sensing systems. Representative polymer- and biofunctionalized modifiers applied to GCE-based sensors for heavy metal detection are summarized in Table 5.

More advanced hybrid architectures have been developed to further improve electrochemical performance, particularly for the detection of toxic heavy metal ions at ultratrace concentrations. Notable examples include mesoporous silica–L-cysteine composites, such as SBA-15/L-cysteine and MCM-41/L-cysteine [85]. In these systems, the ordered mesoporous silica framework provides a large accessible surface area and well-defined mass transport channels, promoting efficient analyte diffusion and signal amplification. Concurrently, L-cysteine introduces strong metal-binding capability through its thiol and amine groups. The synergistic integration of structural porosity and targeted chemical recognition enables effective preconcentration of metal ions within the electroactive region, resulting in significantly reduced detection limits for Cd(II) and Pb(II).

**Figure 7:**
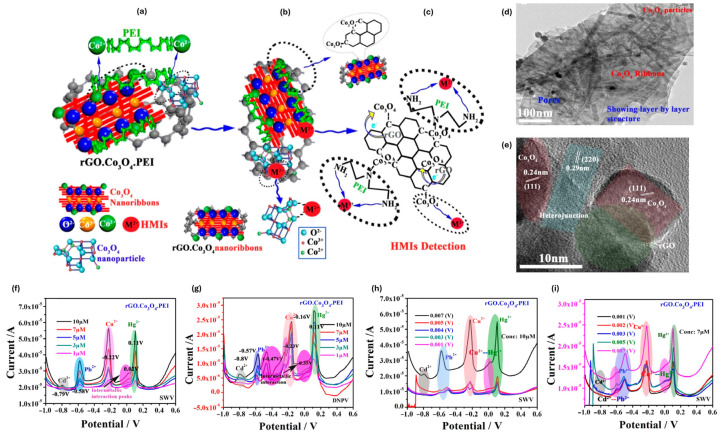
Schematic illustration of the HMI detection mechanism of the rGO·Co_3_O_4_·PEI nanocomposite, highlighting (**a**) coordination interactions between PEI and Co(II) ions, (**b**) association of HMIs with Co_3_O_4_ nanoparticles, and (**c**) synergistic interactions among PEI, rGO, and Co_3_O_4_ during HMI sensing. TEM images of the rGO·Co_3_O_4_·PEI nanocomposite showing (**d**) its porous morphology and (**e**) structural heterogeneity. (**f**–**i**) Proposed influence of HMIs on the electrochemical response, illustrating changes in SWV peaks obtained from SWV and DNPV analyses of the rGO·Co_3_O_4_·PEI nanocomposite. Adapted with permission from reference [82]. Copyright (2022) the authors, some rights reserved; exclusive licensee American Chemical Society. Distributed under a Creative Commons Attribution 4.0 International License (CC BY 4.0).

Expanding upon these hybrid strategies, the controllable synthesis of porous polyethyleneimine (PEI)-functionalized Co_3_O_4_/reduced graphene oxide nanocomposites (rGO-Co_3_O_4_ · PEI) has demonstrated exceptional performance for the simultaneous and individual detection of Cd(II), Pb(II), Cu(II), and Hg(II) [82]. As illustrated in Figure 7, the sensing mechanism is driven by the high density of primary and secondary amino groups on the PEI chains, which serve as highly efficient coordination sites for heavy metal ions. The integration of these polymeric sites with a porous Co_3_O_4_ nanostructure and conductive rGO sheets creates a synergistic environment: the rGO provides a high-speed electron transfer pathway, while the porous Co_3_O_4_ nanoribbons/nanoparticles increase the electroactive surface area and facilitate mass transport. This architecture achieves nanomolar detection limits and maintains high sensitivity even in complex individual or simultaneous analytical scenarios.

Among the most sensitive polymer-based platforms reported is the chitosan-mediated bimetallic Fe–Al mixed metal oxide nanocomposite electrode (Ch-MMON/GCE), which achieved ultralow detection limits of 0.00061 nM for Cd(II), 0.00016 nM for Pb(II), and 0.00026 nM for Hg(II) [88]. The exceptional sensitivity of this system arises from the synergistic interaction between the bimetallic oxide component, which enhances redox activity and increases the density of electroactive sites, and chitosan, which functions both as a stabilizing matrix and as an efficient chelating agent. The abundant amino and hydroxyl groups of chitosan promote strong coordination with metal ions, enabling effective accumulation and pronounced signal enhancement.

Collectively, these studies underscore the critical role of rational surface functionalization and modifier selection in achieving ultrasensitive and selective electrochemical detection of heavy metal ions. By combining conductive polymers for efficient charge transport with bio-inspired modifiers that provide targeted chemical or biological recognition, advanced sensing platforms can be engineered with superior sensitivity, selectivity, and durability. Such hybrid systems offer a robust and adaptable foundation for next-generation electrochemical sensors aimed at environmental monitoring and public health protection.

## 4. Conclusions and Future Outlook

Recent advances in nanomaterial-modified glassy carbon electrodes have substantially expanded the capabilities of electrochemical platforms for simultaneous multi-ion heavy metal detection. The strategic integration of high-surface-area metal–organic frameworks, metal nanoparticles, and conductive carbon nanostructures has enhanced electron-transfer kinetics while increasing the density of accessible coordination sites. These developments have enabled sub-nanomolar detection of environmentally and biologically relevant metal ions, including Pb(II), Cd(II), Cu(II), Zn(II), and Hg(II). As a result, GCE-based sensors are emerging as cost-effective and portable alternatives to established laboratory-bound techniques such as inductively coupled plasma mass spectrometry and atomic absorption spectroscopy, particularly for decentralized and on-site monitoring. Their reliable performance in mildly acidic media further supports their suitability for environmental applications, where metal ion solubility and mobility are optimized.

Beyond materials engineering, recent progress highlights the increasing importance of data-driven signal processing in overcoming intrinsic limitations of multi-ion electrochemical systems. In complex matrices, overlapping stripping peaks often restrict analytical resolution even when advanced nanomaterials are employed. Integrating computational analytics with electrochemical measurements has therefore emerged as a powerful complementary strategy. For example, Leon-Medina et al. combined square-wave voltammetry with dimensionality reduction and machine learning algorithms to classify As(III), Pb(II), and Cd(II) with over 98% accuracy, demonstrating that multivariate analysis can successfully resolve electrochemical responses that are difficult to separate through electrode design alone [100]. Such findings illustrate how coupling nanostructured interfaces with advanced signal analytics can significantly enhance selectivity and robustness in multi-metal detection.

Despite these advances, several challenges remain before large-scale real-world deployment can be achieved. Multi-ion systems are inherently susceptible to interference arising from competitive adsorption, intermetallic formation, and surface site saturation. As the number of analytes increases, these coupled thermodynamic and kinetic interactions frequently lead to peak broadening, signal suppression, and reduced quantitative accuracy. These limitations become more pronounced in complex matrices, including industrial effluents, natural waters, and biological fluids, where organic matter, coexisting ions, and electrode fouling introduce additional variability that is insufficiently addressed in many proof-of-concept demonstrations.

Future progress will require a transition from incremental material optimization toward integrated, mechanism-guided sensor design. Advanced surface engineering approaches, such as ion-imprinted polymers and rationally designed MOFs with size- and coordination-selective binding domains, offer promising routes to improving analyte specificity and mitigating competitive adsorption. In parallel, systematic integration of chemometric tools and machine learning algorithms for automated peak recognition and signal deconvolution will be essential for reliable multi-analyte quantification under realistic conditions. Hybrid sensing architectures that combine electrochemical detection with complementary optical or spectroscopic readouts may further enhance analytical reliability through cross-validation strategies.

Importantly, accumulating theoretical and experimental evidence indicates that electrochemical performance in multi-ion systems is governed primarily by atomic-level interactions between metal ions and interfacial functional groups rather than by bulk conductivity alone. Adsorption energy, coordination geometry, and interfacial charge redistribution dictate preconcentration efficiency and stripping behavior, while competitive adsorption controls peak discrimination. Although numerous nanomaterials have demonstrated enhanced sensitivity, systematic structure–performance correlations remain limited. Bridging this gap will require integrating advanced structural characterization techniques with density functional theory and data-driven modeling to enable predictive, mechanism-based electrode design.

Equally critical is rigorous evaluation of long-term operational stability, reproducibility, and resistance to fouling under continuous or repeated-use conditions—factors that are essential for practical in situ monitoring but remain underexplored. Looking forward, continued advances in electrode miniaturization, low-power electronics, wireless communication, and Internet-of-Things (IoT) integration are expected to yield autonomous, smart GCE-based sensing platforms capable of delivering real-time environmental and health-related data. Through the combined evolution of rational interface engineering, computational analytics, and intelligent system integration, electrochemical multi-ion sensing is poised to transition from a laboratory-scale analytical method to a scalable and impactful technology for environmental protection and public health surveillance.

## Figures and Tables

**Table 1:** Comparative summary of electrochemical techniques used for simultaneous detection of heavy metals.

Technique	Mechanism	Advantages	Disadvantages
Square-Wave Voltammetry (SWV)	Measures current response to a square-wave potential superimposed on a staircase waveform	Fast analysis; good signal-to-noise ratio Low detection limits	Less selective than SWASV Interference from background currents
Square-Wave Anodic Stripping Voltammetry (SWASV)	Preconcentration of metal ions onto the electrode surface followed by stripping using square-wave excitation	High sensitivity; suitable for trace-level detection Short analysis time	Requires optimized deposition parameters Susceptible to electrode fouling
Differential Pulse Voltammetry (DPV)	Applies a series of potential pulses with increasing baseline Measures current difference	Improved resolution between closely spaced peaks Good sensitivity	May suffer from baseline drift Lower sensitivity compared to stripping methods
Differential Pulse Anodic Stripping Voltammetry (DPASV)	Combines metal ion preconcentration on electrode surface with pulse-based stripping	Excellent sensitivity Allows simultaneous multi-metal detection	Sensitive to experimental conditions Prone to matrix effects
Differential Pulse Cathodic Stripping Voltammetry (DPCSV)	Involves formation of insoluble metal complexes on electrode surface, followed by cathodic reduction during potential sweep	High sensitivity for certain an-ionic metal species (e.g., Cr(VI)) Selective for species reducible at cathodic potentials	Limited to metals forming stable adsorbed complexes Requires careful pH and ligand control
Differential Pulse Adsorptive Stripping Voltammetry (DSAdSV)	Involves adsorption of metal–ligand complexes onto electrode surface followed by stripping	Very low detection limits Suitable for metals forming stable complexes	Requires suitable complexing agents Complex optimization

**Table 2:** Summary of carbon nanostructure-driven composites modifiers integrated with glassy carbon electrodes for simultaneous detection of heavy metal ions in aqueous media.

Modifiers	Electrolyte	Detection Technique	Deposition Time (s)	LOD (Metals)	Linear Range	Real Sample	Ref.
p(Cys).gCN/GCE	0.1 M PBS (pH = 7.4)	SWV		4.9 nM (Cd(II)) 18.6 nM (Zn(II))	1–100 µM (Cd(II)) 5–100 µM (Zn(II))	white rice, black tea, red pepper powder, bottled drinking water, and marine salt	[43]
MgFe-LDH/graphene/GCE	0.1 M ABS (pH = 4.5)	SWASV	180	5.9 nM (Cd(II)) 2.7 nM (Pb(II))	0.1–1.0 µM (Cd(II)) 0.1–1.0 µM (Pb(II))	lake and tap water	[44]
BMIMPF_6_–MWCNTs/GCE	0.1 M ABS	SWASV	360 (Cd(II)) 300 (Pb(II))	4.44 nM (Cd(II)) 4.35 nM (Pb(II))	22–600 µg L^−1^ (Cd(II)) 5–1850 µg L^−1^ (Pb(II))	bottled water, lake water, tap water, and well water	[22]
rGO-PANi-HBr/GCE	0.01 M PBS (pH = 6.0)	DPASV	60	6.5 nM (Cd(II)) 7.3 nM (Pb(II))	0.01–0.18 µM (Cd(II)) 0.01–0.23 µM (Pb(II))	Tap, mineral, and industrial water; plasma	[45]
rGO-SbNPs/GCE	0.1 M KCl (pH = 3.0)	SWASV		45.3 nM (Cd(II)) 16.5 nM (Pb(II)) 34.1 nM (Cu(II)) 4.8 nM (Hg(II))	0.1–4.5 µM	Drinking water	[46]
pg-C_3_N_4_/CoMn_2_O_4_/GCE	0.1 M ABS (pH = 5.0)	SWASV	180	21.0 nM (Cd(II)) 14.0 nM (Pb(II))	0.5–7.0 µM (Cd(II)) 0.2–4.4 µM (Pb(II))	River, lake, and tap water	[47]
TiO_2_/rGO/GCE	0.1 M BRB (pH = 4.0)	DPASV	120	28.2 nM (Cd(II)) 11.7 nM (Pb(II))	0.044–0.89 µM (Cd(II)) 0.024–0.48 µM (Pb(II))	River water	[48]
Fe_3_O_4_@MPC-2/GCE	0.1 M ABS (pH = 5.0)	DPASV	150	12.1 nM (Pb(II)) 7.8 nM (Hg(II))	0.2–5.0 µM (Pb(II)) 1.0–4.0 µM (Hg(II))	Tap water	[49]
GO/MnO_2_/GCE	0.1 M ABS (pH = 5.0)	SWASV	180	3.33 nM (Pb(II)) 1.67 nM (Cu(II))	0.05–1.0 µM	River water	[26]
(Hg/rGO) film/GCE	0.01 M HNO_3_ 0.02 M KNO_3_ (pH = 2.0)	DPASV	500	1.51 nM (Cd(II)) 0.87 nM (Pb(II)) 10.86 nM (Cu(II))	0.018–0.445 µM (Cd(II)) 0.024–0.48 µM (Pb(II)) 0.031–0.79 µM (Cu(II))		[50]
GO-Fe_3_O_4_-PAMAM/GCE	0.1 M ABS (pH = 4.5)	SWASV	160	0.62 nM (Cd(II)) 0.63 nM (Pb(II))	0.2–140 μg L ^−1^ (Cd(II)) 0.4–120 μg L ^−1^ (Pb(II))	Lake and River water	[51]
Ti_3_C_2_T_x_–rGO/GCE	PBS (pH = 5.0)	DPV	-	0.31 nM (Cd(II)) 0.18 nM (Cu(II))	1–150 nM (Cd(II)) 1–150 nM (Cu(II))	Tap and lake water	[52]
ErGO/GCE	0.1 M ABS (pH = 5.0)	DPASV	-	9.1 nM (Cd(II)) 12.2 nM (Pb(II))	0.089–889 µM (Cd(II)) 0.048–482 µM (Pb(II))	River water	[53]
STB/Gs/GCE	0.1 M ABS (pH = 5.0)	SWASV	150	1.17 nM (Cd(II)) 0.38 nM (Pb(II)) 0.61 nM (Hg(II))	0.25–3 µM (simultaneous) 0.05–5 µM (Cd(II)) 0.025–8.5 µM (Pb(II)) 0.05–7.5 µM (Hg(II))		[54]
GDY/GCE	0.1 M ABS (pH = 6.0)	SWASV	600	0.46 nM (Cd(II)) 1.72 nM (Pb(II))	0.01–1 µM	Lake and tap water	[55]

Note: p(Cys).gCN = poly-cysteine functionalized graphitic carbon nitride; PBS = phosphate-buffer solution; SWV = square wave voltammetry; MgFe-LDH = magnesium–iron layered double hydroxide; ABS = acetate buffer solution; SWASV = square wave anodic stripping voltammetry; BMIMPF_6_ = 1-butyl-3-methylimidazolium hexafluorophosphate (ionic liquid); MWCNTs = multi-walled carbon nanotubes; rGO = reduced graphene oxide; PANi-HBr = polyaniline doped with hydrobromic acid; DPASV = differential pulse anodic stripping voltammetry; SbNPs = antimony nanoparticles; pg-C_3_N_4_ = porous graphitic carbon nitride; CoMn_2_O_4_ = cobalt–manganese oxide; TiO_2_ = titanium dioxide; BRB = Britton–Robinson buffer; Fe_3_O_4_ = magnetite; MPC-2 = mesoporous carbon nanochain; GO = graphene oxide; MnO_2_ = manganese dioxide; HNO_3_ = nitric acid; KNO_3_ = potassium nitrate; PAMAM = poly(amidoamine) dendrimers; Ti_3_C_2_Tx = MXene (titanium carbide with surface terminations); ErGO = electrochemically reduced graphene oxide; STB/Gs = sulfur-doped C_3_N_4_ tube bundles and graphene nanosheets; GDY = graphdiyne.

**Table 3:** Summary of MOF- and COF-based hybrid modifiers integrated with glassy carbon electrodes for simultaneous detection of heavy metal ions in aqueous media.

Modifiers	Electrolyte	Detection Technique	Deposition Time (s)	LOD (Metals)	Linear Range	Real Sample	Ref.
Fe-Co-MOF@MWCNT-COOH/GCE	0.1 M ABS (pH = 5.0)	DPASV	270	23.1 nM (Cd(II)) 12.0 nM (Pb(II))	890–8890 nM (Cd(II)) 97–5000 nM (Pb(II))	Lake and river water	[27]
ZIF-7@PANI/GCE	0.1 M ABS (pH = 5.0)	DPV		2.96 nM (Cd(II)) 10.60 nM (Pb(II))	10–500 (Cd(II)) 20–800 (Pb(II))	tap, mineral, and seawater	[28]
MIL-101(Cr)-(COOH)_2_@ MWCNTs/GCE	0.079 M HNO_3_	DPASV	120	80 nM (Pb(II)) 90 nM (Cu(II)) 40 nM (Hg(II))	0.11–15.4 μM (Pb(II)) 0.11–20.1 (Cu(II)) 0.06–20.1 (Hg(II))		[29]
Hg/CMWCNTs@ZIF-8/GCE	0.1 M ABS (pH = 5.5)	DPCSV	300	102.6 nM (Cu(II)) 79.9 nM (Zn(II))	0.05–1.0 mg L^−1^	Fruit juice beverages	[30]
Bi@ZIF-8/CMWCNTs/GCE	ABS (pH = 4.5)	DPASV	300	7.74 nM (Cd(II)) 3.67 nM (Pb(II))	2.0–50.0 μg L^−1^	Lake, wastewater treatment plant effluent	[31]
ZIF-67/GO/GCE	0.1 M ABS (pH = 5.0)	DPV	-	5 nM (Pb(II)) 2 nM (Hg(II)) 1 nM (Zn(II)) 0.6 nM (Cr^3+^)	0.01–0.17 μM (Pb(II)) 0.03–0.15 μM (Hg(II)) 0.04–0.24 μM (Zn(II)) 0.02–0.22 (Cr^3+^)		[32]
La-doped ZIF-67@L-Cys/GCE	0.1 M PBS (pH = 6.0)	DPV	180	10 nM (Cd(II)) 52 nM (Hg(II))	300 nM–30 μM (Cd(II)) 700 nM–30 μM (Hg(II))	River water	[33]
PAMAM/Ni-MOF/GCE	0.2 M ABS (pH = 5.0)	SWASV	450	5.85 nM (Pb(II)) 12.12 nM (Cu(II))	1–100 μg·L^−1^ (Pb(II))1–100 μg·L^−1^ (Cu(II))	Tap water	[34]
MIL-53(Fe)/Ag_2_CrO_4_/GCE	ABS (pH = 6.14)	DPV	-	27.40 nM (Pb(II)) 86.96 nM (Cu(II))	7.49–320.00 μmol L^−1^ (Pb(II)) 7.49–320.04 μmol L^−1^ (Cu(II))	Lake water	[35]
Cu-MOF	0.1 M KCl	SWASV	400	0.85 nM (Hg(II)) 0.54 nM (Tl^+^)	1–400 ppb (Hg(II)) 0.5–700 ppb (Tl^+^)	Agricultural pool water	[36]
Fc-NH_2_-Ni-MOF/GCE	0.1 M ABS	DPASV	100	7.1 nM (Cd(II)) 0.2 nM (Pb(II)) 6.3 nM (Cu(II))	0.01–2.0 μM (Cd(II)) 0.001–2.0 μM (Pb(II)) 0.01–2.0 μM (Cu(II))	Tap water	[37]
Bi/UiO-66-NH_2_ @CNHs/GCE	0.2 M ABS (pH = 5.0)	DPASV	600	10.56 nM (Cd(II)) 6.65 nM (Pb(II))	0.20–0.80 μM (Cd(II)) 0.20–0.80 μM (Pb(II))	Tap and lake water	[38]
Cr-BDC/GCE	0.2 M ABS (pH = 5.0)	DPASV	150	0.19 nM (Cd(II)) 0.12 nM (Pb(II)) 0.12 nM (Hg(II))	0–10 nM	Tap water	[39]
2D COF_BTLP-1_/GCE	0.2 M ABS (pH = 4.8) (N_2_-saturated)	DPASV	250	44.5 nM (Cd(II)) 22.5 nM (Pb(II)) 66.0 nM (Cu(II)) 29.2 nM (Hg(II))	0.135–7.00 μM (Cd(II)) 0.07–7.00 μM (Pb(II)) 0.200–7.00 μM (Cu(II)) 0.09–7.00 μM (Hg(II))		[40]
TPT-COF/GCE	universal buffer solution (pH = 2.7)	SWASV	230	1.8 nM (Cd(II)) 1.1 nM (Pb(II))	5–300 nM (Cd(II)) 3–110 nM (Pb(II))	White rice, green tea, drinking water, marine salt, sugar, black tea, dried powdered spices	[41]
SNW_1_/GCE	0.1 M solution of (KNO_3_ + 0.01 M HCl) (pH = 2.0)	SWASV	150	0.72 nM (Pb(II)) 12.11 nM (Hg(II))	0.01–0.3 μM (Pb(II)) 0.05–0.3 μM (Hg(II))	Black tea, red pepper, white rice	[42]

Note: Fe-Co-MOF = iron–cobalt metal–organic framework; MWCNT-COOH = acid-functionalized multi-walled carbon nanotubes; ZIF = zeolitic imidazolate framework; PANI = polyaniline; MIL-101(Cr)-(COOH)_2_ = chromium-based MOF functionalized with dicarboxylate groups; CMWCNTs = carboxylated multi-walled carbon nanotubes; GO = graphene oxide; L-Cys = L-cysteine; PBS = phosphate-buffer solution; PAMAM = poly(amidoamine) dendrimers; MIL-53(Fe) = iron-based MOF; Ag_2_CrO_4_ = silver chromate; Cu-MOF = copper-based MOF; Fc-NH_2_-Ni-MOF = ferrocene-functionalized Ni(II)-based MOF; UiO-66-NH_2_ = zirconium-based MOF functionalized with amino groups; CNHs = carbon nanohorns; Cr-BDC = chromium terephthalate MOF; 2D COFBTLP-1 = 2D covalent organic framework synthesized via triformylbenzene and benzene dithiol linkers; TPT-COF = triazine–phenyl–triazine covalent organic framework; SNW1 = Schiff base network-1 covalent organic framework; ABS = acetate buffer solution; HNO_3_ = nitric acid.

**Table 4:** Summary of metal/metal oxide modifiers integrated with glassy carbon electrodes for simultaneous detection of heavy metal ions in aqueous media.

Modifiers	Electrolyte	Detection Technique	Deposition Time (s)	LOD (Metals)	Linear Range	Real Sample	Ref.
GCE-BFS	ABS (pH 5.0) with 0.1 M KNO_3_ solution	DPV	70	84 nM (Pb(II)) 440 nM (Cu(II))	0.5–80 μM		[56]
Bi/CP/GCE	0.1 M ABS	SWASV	15	222 nM (Cd(II)) 48.3 nM (Pb(II))	50–500 μg L^−1^ (Cd(II)) 25–500 μg L^−1^ (Pb(II))	Tap water	[57]
BiFE/GCE	0.1 M ABS (pH = 5.0)	DPASV	110	8.27 nM (Cd(II)) 3.14 nM (Pb(II)) 16.4 nM (Zn(II)) 14.8 nM (Cu(II))	5.0–110.0 ppb		[58]
Sphere-BiNPs/GCE	0.1 M ABS (pH = 4.5)	SWASV	240	14.2 nM (Cd(II)) 7.7 nM (Pb(II)) 61.2 nM (Zn(II))	20–130 μg·L^−1^ (Zn(II)) 10–60 μg·L^−1^ (Cd(II)) 6–54 μg·L^−1^ (Pb(II))	Seawater	[59]
BiFGCE	0.1 M ABS	SWASV		3.56 nM (Cd(II)) 0.97 nM (Pb(II))		Tap water	[60]
BiVO_4_-NS/GCE	0.1 M HEPES buffer (pH = 8)	SWASV	0.2	2750 nM (Cd(II)) 2320 nM (Pb(II)) 2720 nM (Cu(II)) 1200 nM (Hg(II))	0–110 μM		[61]
Fe_2_O_3_/Bi_2_O_3_/GCE	0.1 M ABS (pH = 5.0)	SWASV	300	0.56 nM (Cd(II)) 0.36 nM (Pb(II))	0.002–4.0 μM	Lake water and milk	[62]
Bi/GDY/GCE	0.01 M ABS (pH = 6.0)	DPASV	300	0.17 nM (Cd(II)) 0.15 nM (Pb(II)) 0.12 nM (Hg(II))	10.0 nM −100.0 μM	Seawater	[63]
Bi_2_O_3_@NPBi/GCE	0.1 M ABS (pH = 4.0)	SWASV	180	0.27 nM (Cd(II)) 0.10 nM (Pb(II))	0.5–200 µg/L	Tap water	[64]
Sb/Bi-GCE	0.1 M ABS (pH = 4.5)	DPASV	100	4.45 nM (Cd(II)) 0.05 nM (Pb(II))	1–100 ppb (Cd(II)) 0.1–100 ppb (Pb(II))	Soil and tap water	[65]
Co_3_O_4_-NC/rGO/GCE	0.1 M ABS with 0.05 M KCl solution	SWASV	250	0.55 nM (Cd(II)) 0.16 nM (Pb(II))	0.1–450 ppb	Agricultural pool water, tap water, well water	[66]
CeCo_3_O_6_/Nafion/GCE	0.1 M ABS	DPV	240	214 nM (Cd(II)) 205 nM (Cu(II))	0.5–5 mg/L	Tap water	[67]
Co_3_O_4_/GO/Nafion/GCE	0.1 M ABS (pH = 4.5)	DPASV	250	57 nM (Cd(II)) 29.4 nM (Pb(II))	0.1–450 ppb	Lotus pool	[68]
CoFe_2_O_4_@CTS/GCE	0.1 M ABS (pH = 5.0)	DPAdSV	90	2.76 nM (Cd(II)) 0.19 nM (Pb(II))	2.50–30.7 μg L^−1^	Fresh water	[69]
Ag-HG/GCE	ABS (pH = 4.0)	SWASV	280	200 nM (Cd(II)) 390 nM (Pb(II)) 350 nM (Cu(II)) 230 nM (Hg(II))	1.0–10 μM	River water	[70]
TiO_2_ NF/AgCNF/Nafion/GCE	0.1 M ABS	SWASV	600	40.14 nM (Cd(II)) 31.69 nM (Pb(II)) 93.31 nM (Cu(II)) 208.26 nM (Hg(II))	0.2–1.2 µM		[71]
OL-MBene@GCE	ABS (pH = 4.0)	SWASV	100	112 nM (Cd(II)) 48.1 nM (Pb(II)) 49.5 nM (Cu(II)) 67.8 nM (Hg(II))	0.5–1.9 µM (Cd(II)) 0.1–0.9 µM (Pb(II)) 0.5–1.2 µM (Cu(II)) 0.5–1.7 µM (Hg(II))	Tap water	[72]
AGCE/GCE	0.2 M ABS (pH = 4.5)	DPV	900	17 nM (Cd(II)) 0.3 nM (Pb(II))	0.05–5 µM	Lake water	[73]
ZnO-NP-Nafion/GCE	0.1 M ABS (pH = 4.6)	SWASV		16.21 nM (Cd(II)) 11.88 nM (Pb(II)) 11.59 nM (Fe^3+^) 47.33 nM (Cu(II))		Drilling water	[74]
L-Cys/Ag@MnO_2_/GCE	M ABS (pH = 5.0)	SWASV	120	0.07 nM (Cd(II)) 0.05 nM (Pb(II))	0.005–0.1 μM	Tap water	[75]

Note: BFS = blast furnace slag; ABS = acetate buffer solution; DPV = differential pulse voltammetry; Bi/CP = bismuth–carboxyphenyl composite; SWASV = square wave anodic stripping voltammetry; BiFE = bismuth film electrode; DPASV = differential pulse anodic stripping voltammetry; Sphere-BiNPs = spherical bismuth nanoparticles; BiFGCE = bismuth film glassy carbon electrode; BiVO_4_-NS = bismuth vanadate nanospheres; HEPES = 4-(2-hydroxyethyl)-1-piperazineethanesulfonic acid; Fe_2_O_3_ = hematite; Bi_2_O_3_ = bismuth oxide; GDY = graphdiyne; Bi_2_O_3_@NPBi = nanoporous bismuth decorated with bismuth oxide; Sb/Bi-GCE = antimony–bismuth modified GCE; Co_3_O_4_-NC = cobalt oxide nanocrystal; rGO = reduced graphene oxide; KCl = potassium chloride; CeCo_3_O_6_ = cerium–cobalt oxide composite; Nafion = sulfonated tetrafluoroethylene-based polymer; CoFe_2_O_4_@CTS = cobalt ferrite nanoparticles stabilized with chitosan; DPAdSV = differential pulse adsorptive stripping voltammetry; Ag-HG = silver hydrogel; TiO_2_ NF = titanium dioxide nanofibers; AgCNF = silver-coated carbon nanofibers; OL-MBene = open-layered metal boride (MXene-like material); AGCE = acid phosphate-activated glassy carbon electrode; ZnO-NP = zinc oxide nanoparticles; L-Cys = L-cysteine; MnO_2_ = manganese dioxide.

**Table 5:** Summary of polymer- and biofunctionalized modifiers integrated with glassy carbon electrodes for simultaneous detection of heavy metal ions in aqueous media.

Modifiers	Electrolyte	Detection Technique	Deposition Time (s)	LOD (Metals)	Linear Range	Real Sample	Ref.
Hg-Bi/PDAAQ/GCE	0.1 M ABS (pH = 5.0)	SWASV	120	107 nM (Cd(II)) 3.18 nM (Pb(II)) 37 nM (Zn(II))	0–50 μg/L	Tap water	[76]
4-ATP-DPPH/GCE	0.1 M ABS (pH = 4.5) (deoxygenated)	DPV	180	13.35 nM (Cd(II)) 5.79 nM (Pb(II))	2.5–400 μg L^−1^	Spring and Tap water; Apple and Grape pomace extract	[77]
Cl-DPTU/GCE	BRB (pH 4)	SWASV	100	6.45 nM (Cd(II)) 11.0 nM (Pb(II)) 7.85 nM (Cu(II)) 9.15 nM (Hg(II)) 16.9 nM (Zn(II)) 178.8 nM (Sr(II))	2 nM–10 µM (Zn(II)) 1 nM–5 µM (Cd(II)) 1 nM–5 µM (Pb(II)) 1.1 nM–10 µM (Cu(II)) 1 nM–5 µM (Hg(II)) 8 nM–5 µM (Sr(II))	Tap and drinking water	[78]
PA-Si-SA/GCE	0.1 M ABS (pH = 5.6)	DPAdSV	180	4.1 nM (Cd(II)) 24.0 nM (Pb(II))	0.05–2.0 µM	Tap water, drinking water, artificial lake water	[79]
PAn-RB3R/GCE	0.1 M ABS (pH = 5.0)	DPV	240	1.6 nM (Pb(II)) 0.011 nM (Hg(II))	1–22 µM		[80]
NG-HgFE/GCE	0.1 M ABS (pH = 5.0)	SWASV	60	2.22 nM (Cd(II)) 1.26 nM (Pb(II))	0.5–12.0 µg L^−1^	Seawater	[81]
rGO·Co_3_O_4_·PEI NCP/GCE	5 mM Fe(CN)_6_^3−^/^4−^ + 1 M KCl (pH 5.0, NaOAc–HOAc)	DNPV	-	1.07 nM (Cd(II)) 0.29 nM (Pb(II)) 2.40 nM (Cu(II)) 1.12 nM (Hg(II))	1–10 µM		[82]
PAni–RYFG/GCE	ABS (pH = 5.0)	DPV	-	6.2 nM (Pb(II)) 2.0 nM (Hg(II))	1–21 μM	Lake water, groundwater	[83]
PAni-Bi NPs@GO-MWCNT/GCE	0.01 M PBS (pH = 7.2)	DPV		0.50 nM (Cu(II)) 0.01 nM (Hg(II))	0.5 nmol/L–5 mmol/L (Cu(II)) 0.01 nmol/L–5 mmol/L (Hg(II))		[84]
SBA-15/L-Cys/GCE	ABS (pH = 4.2)	SWASV	300	1.96 nM (Cd(II)) 1.74 nM (Pb(II))	5–80 µg L^−1^	Tap water	[85]
MCM-41/L-cys/GCE	ABS (pH = 4.2)	SWASV	300	2.05 nM (Cd(II)) 3.67 nM (Pb(II))	10–80 µg L^−1^	Tap water	[85]
nc-Chi/GCE	0.1 M ABS (pH = 4.5)	SWASV	180	19.1 nM (Cd(II)) 4.3 nM (Pb(II)) 57.3 nM (Cu(II)) 43.1 nM (Zn(II))	0.1–0.9 µM (Cd(II)) 0.040–0.400 µM (Pb(II)) 0.1–0.9 µM (Cu(II)) 0.1–0.9 µM (Zn(II))	Tap water	[86]
SWCNT/L-Cys/Nafion-IL/GCE	0.1 M ABS (pH = 5.0)	SWASV	120	0.45 nM (Cd(II)) 0.39 nM (Pb(II))	5–50 μg L^−1^		[87]
Ch-MMON/GCE	0.1 M ABS (pH = 4.5)	DPV	300	6.1 × 10^−4^ nM (Cd(II)) 1.6 × 10^−4^ nM (Pb(II)) 2.6 × 10^−4^ nM (Hg(II))	5–125 ppt	River water	[88]

Note: Hg-Bi/PDAAQ = bismuth–mercury composite with poly(1,2-diaminoanthraquinone); ABS = acetate buffer solution; 4-ATP-DPPH = 4-aminothiophenol with 1,1-diphenyl-2-picrylhydrazyl radicals; Cl-DPTU = 1-(3-chlorophenyl)-3-dodecanoylthiourea; BRB = Britton–Robinson buffer; PA-Si-SA = polyamide/silica/sodium alginate composite; PAn-RB3R = polyaniline–Reactive Blue 3R composite; NG-HgFE = Nafion–guanine-coated mercury film electrode; rGO·Co_3_O_4_·PEI NCP = reduced graphene oxide with cobalt oxide and polyethylenimine nanocomposite; DNPV = differential normal pulse voltammetry; PAni–RYFG = polyaniline–Reactive Yellow FG dye composite; PAni-Bi NPs@GO-MWCNT = polyaniline doped with bismuth nanoparticles on graphene oxide–MWCNT hybrid; PBS = phosphate-buffer solution; SBA-15 = Santa Barbara Amorphous-15 mesoporous silica; MCM-41 = Mobil Composition of Matter No. 41 mesoporous silica; L-Cys = L-cysteine; nc-Chi = natural clay–chitosan composite; SWCNTs = single-walled carbon nanotubes; Nafion-IL = Nafion–ionic liquid composite; Ch-MMON = chitosan-mediated mixed metal oxide nanocomposite.

## Data Availability

The original contributions presented in the study are included in the article, further inquiries can be directed to the corresponding authors.
